# Association between hyperlipidemia and trigger finger: A nationwide population-based cohort study

**DOI:** 10.1371/journal.pone.0288426

**Published:** 2023-07-10

**Authors:** Pei-Tsen Chen, Han-Wei Zhang, Zhi-Ren Tsai, Hsiao-Ching Peng, Yu-Shan Lin, Jeffrey J. P. Tsai, Chao-Wen Lin

**Affiliations:** 1 Department of Physical Medicine and Rehabilitation, Cardinal Tien Hospital, New Taipei, Taiwan; 2 Department of Anatomy and Cell Biology, College of Medicine, National Taiwan University, Taipei, Taiwan; 3 Biomedica Corporation, New Taipei, Taiwan; 4 Ph.D Program for Aging, China Medical University, Taichung, Taiwan; 5 Institute of Population Health Sciences, National Health Research Institutes, Miaoli, Taiwan; 6 Department of Electrical and Computer Engineering, Institute of Electrical Control Engineering, National Yang Ming Chiao Tung University, Hsinchu, Taiwan; 7 Department of Computer Science & Information Engineering, Asia University, Taichung, Taiwan; 8 Department of Medical Research, China Medical University Hospital, China Medical University, Taichung, Taiwan; 9 Center for Precision Medicine Research, Asia University, Taichung, Taiwan; 10 Department of Bioinformatics and Medical Engineering, Asia University, Taichung, Taiwan; 11 Department of Ophthalmology, National Taiwan University Hospital, Taipei, Taiwan; AIIMS: All India Institute of Medical Sciences, INDIA

## Abstract

The cause of trigger fingers remains uncertain. High lipid levels in the blood may reduce blood supply to the distal fingers and promote inflammation. We aimed to explore the association between hyperlipidemia and trigger finger. A nationwide population-based cohort study using longitudinal data from 2000 to 2013, 41,421 patients were included in the hyperlipidemia cohort and 82,842 age- and sex-matched patients were included in the control cohort. The mean age was 49.90 ± 14.73 years in the hyperlipidemia cohort and 49.79 ± 14.71 years in the control cohort. After adjusting for possible comorbidities, the hazard ratio of trigger finger in the hyperlipidemia cohort was 4.03 (95% confidence interval [CI], 3.57–4.55), with values of 4.59 (95% CI, 3.67–5.73) and 3.77 (95% CI, 3.26–4.36) among male and female patients, respectively. This large-scale population-based study demonstrated that hyperlipidemia is correlated to trigger finger.

## Introduction

Trigger finger, also known as stenosing tenosynovitis, is a common disease among people aged 50 to 60 years [1, 2]. The prevalence of trigger finger is about 3% in the general population [3]. The manifestations of trigger finger include pain and locking or tightness at the base of one or more fingers. The thumb and fourth finger are most frequently involved [4]. Patients often complain of a popping or catching sensation during finger extension from the flexion position. In this condition, the flexor tendon cannot glide smoothly within the tendon sheath, and sometimes a palpable nodule is noted on physical examination. Diagnosis can be made according to the clinical symptoms and signs, or the findings on ultrasound examination. On ultrasound, the flexion digitorum tendon appears to be stuck in the swollen annular pulley. Although trigger finger is not a life-threatening disease, it limits the ability to perform hand activities and greatly affects the quality of life.

The etiology of trigger finger is still uncertain. Repeated hand work and exposure to tools that generate vibrations are thought to be related to trigger finger [5, 6]. However, one study showed no association between trigger finger and activities at the work place [7]. According to previous studies, female sex, increasing age, diabetes mellitus, obesity, rheumatoid arthritis, hypothyroidism and carpal tunnel syndrome are possible risk factors for trigger finger [6, 8, 9]. In general practice, clinicians seldom check blood examination findings in patients with trigger finger, which may lead to poor control of underlying diseases and a high trigger finger recurrence rate.

Hyperlipidemia is a metabolic disorder that occurs frequently in middle-aged people. A high lipid level in blood may cause the impairment of microcirculation and increase the accumulation of reactive oxygen species. Moreover, it may decrease distal finger blood supply, decrease cell viability, and incite inflammatory reactions [10]. Nevertheless, limited studies have reported the relationship between hyperlipidemia and trigger finger. Thus, this population-based cohort study was conducted in Taiwan to elucidate whether hyperlipidemia is associated with trigger finger.

## Materials and methods

### Data source

The National Health Insurance Research Database (NHIRD), founded in 1996 in Taiwan, includes all medical histories and insurance claims data from the National Health Institute (NHI) database of 22.96 million persons (99% of Taiwan’s population) covered by the universal health insurance program [11]. The NHIRD has data on real-world practice outcomes and has been primarily recognized for its significance and clinical influence beyond the results of clinical trials performed at numerous centers that assist in the development of clinical practice recommendations for illness care [12]. To deter medical fraud, the NHI maintains strict record-keeping standards and can impose fines of 100 times the healthcare expenditure. Thus, the NHIRD provides trustworthy healthcare data based on large health data analytics for real-world proof. The database tracks all patients, using the International Classification of Disease, 9th Revision, Clinical Modification (ICD-9-CM).

The Taiwanese Longitudinal Health Insurance Database (LHID) within the NHIRD provides claims data for 1 million randomly sampled beneficiaries from 1996 to 2013. NHIRD data dependability has been improved by considering 2000–2013. The Institutional Review Board of Cardinal Tien Hospital (CTH-111-3-5-027) approved the study. Deidentified/anonymized database data allowed the Institutional Review Board to waive the requirement for informed consent. All study outcomes were considered. The authors ensure that the data and conclusions are presented objectively and in an unbiased manner. All data and related metadata underlying reported findings have been deposited in the public data repository.

### Study design and population

**Fig 1:**
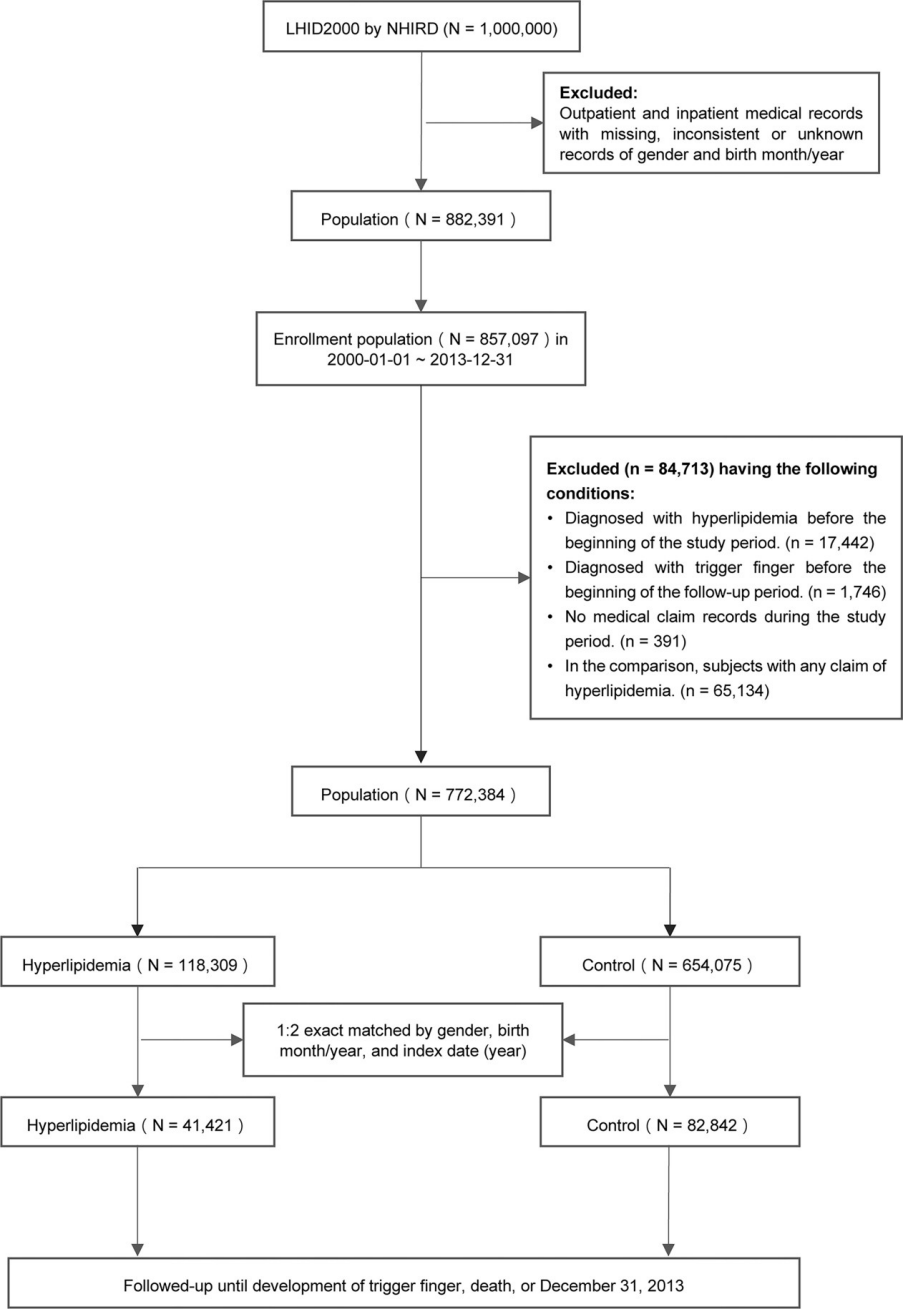
Flow of the study population.

This retrospective cohort analysis used LHID inpatient and outpatient claims data. We removed LHID beneficiaries with missing gender and birth month/year records. Then, 857,097 people remained between 2000 and 2013. Among these people, 84,713 were removed because (1) they were diagnosed with hyperlipidemia before the study began, (2) they had trigger finger before the follow-up period, (3) they had no medical claims data throughout the follow-up period, and (4) there was any claim of hyperlipidemia in the control cohort. Finally, this study considered 772,384 people. Fig 1 depicts the research population selection.

### Selection and measurement of exposure

We identified hyperlipidemia cases (ICD-9-CM codes 272.0–272.4) from 2000 to 2013 in the included population. The diagnosis of hyperlipidemia was made if serum total cholesterol>200 mg/dL or triglyceride>150 mg/dL. Hyperlipidemia cohort members had at least three outpatient visits or one hospitalization for hyperlipidemia. The control cohort was from the same dataset without hyperlipidemia. Exact matching in a ratio of 1:2 by birth year and month, sex, and index year created this cohort. Both cohorts recruited 124,263 people, including 41,421 hyperlipidemia patients and 82,842 controls. For all subsequent analyses, the index date for the hyperlipidemia cohort was the first hospitalization or outpatient clinic visit associated with a hyperlipidemia diagnosis and that for the control cohort was the first claim record date in the study period. The study participants were followed from then until 2013.

### Definition of the incidence of trigger finger

Trigger finger incidence was represented as occurrences per 10,000 person-years for the hyperlipidemia and control cohorts. The primary outcome measure in this study was a new trigger finger diagnosis based on ICD-9-CM code 727.03 in at least three outpatient medical claims (to reduce inadvertent miscoding in outpatient reimbursement data) or one inpatient claim. For all subsequent analyses, the trigger finger diagnostic date was the first inpatient or outpatient clinic attendance date. Trigger finger diagnosis, death, or December 31, 2013 (the final date of observation) was the endpoint of follow-up from the index date.

### Comorbidities

The LHID used ICD-9-CM codes to assess illness conditions. The following comorbidities were considered in the present study according to previous studies [6, 9, 13–19]: diabetes mellitus (ICD-9-CM code 250), rheumatoid arthritis (ICD-9-CM code 714.0), hypertension (ICD-9-CM code 401), hypothyroidism (ICD-9-CM code 244.0), hyperthyroidism (ICD-9-CM code 242.9), gout (ICD-9-CM code 274.9), osteoarthritis (ICD-9-CM code 715.9), depression (ICD-9-CM code 296.2), carpal tunnel syndrome (ICD-9-CM code 354.0), distal radial fracture (ICD-9-CM code 813.42), cardiovascular disease (ICD-9-CM code 429.2) and obesity (ICD-9-CM codes 278.00–278.03, V77.8). To reduce data selection bias, these concomitant illnesses were identified and characterized based on a minimum of three outpatient visits or one hospitalization before trigger finger diagnosis. We considered highly imbalanced comorbidities in models of subsequent analysis to avoid bias.

### Statistical analysis

For continuous and categorical variables, descriptive statistics are reported as mean ± standard deviation and frequency with percentage (%). The demographics and comorbidities of the matched control and hyperlipidemia populations were compared using the chi-squared test and Student’s *t* test. The Cox proportional hazards regression model assessed trigger finger risk hazard ratios (HRs) with 95% confidence intervals (CIs) between cohorts. Adjusting for age, sex, urbanization, insurance amount, and comorbidities, we calculated the independent impact of hyperlipidemia on the incidence of trigger finger. We also evaluated gender differences in stratified analysis. The Kaplan–Meier analysis and log-rank test were used to determine the connection between hyperlipidemia and trigger finger. All analyses were performed using the MetaTrial Platform (Biomedica Corporation, Taiwan, Taipei) and Statistical Product and Service Solutions (SPSS; Version 22; IBM Corp, Armonk, NY). All statistical tests were two-sided, and the significant level was set at 0.05.

## Results

### Characteristics of the study population

After patient matching, the hyperlipidemia and control cohorts included 41,421 and 82,842 people, respectively. Table 1 shows the demographics and comorbidities for both cohorts. The control cohort had a mean age of 49.79 ± 14.71 years, while the hyperlipidemia cohort had a mean age of 49.90 ± 14.73 years. The two cohorts had significantly different distributions for the urbanization level and insurance amount. Moreover, numerous comorbidities significantly differed between the hyperlipidemia and control cohorts, with the hyperlipidemia cohort having greater comorbidities, as expected. Among patients in hyperlipidemia group (n = 41,421), 16,309 patients (39.37%) took medications for hyperlipidemia.

**Table 1:** Characteristics of the hyperlipidemia and control cohorts.

Characteristic	Control cohort (n = 82,842)	Hyperlipidemia cohort (n = 41,421)	*P* value
**Age**, years	49.79 ± 14.71	49.90 ± 14.73	0.211
**Sex**			matched
Male	43,908 (53.00)	21,954 (53.00)	
**Urbanization level** ^a^			< 0.001
1 (highest)	42,914 (51.80)	22,201 (53.60)	
2	25,830 (31.18)	12,336 (29.78)	
3	5,683 (6.86)	2,670 (6.45)	
4 (lowest)	897 (1.08)	450 (1.09)	
Unknown	7,518 (9.08)	3,764 (9.09)	
**Insurance amount**^b^, NT$			< 0.001
Financially dependent	561 (0.68)	334 (0.81)	
1–19999	34,078 (41.14)	13,087 (31.60)	
20000–39999	30,811 (37.19)	16,996 (41.03)	
≥40000	12,531 (15.13)	8,752 (21.13)	
Unknown	4,861 (5.87)	2,252 (5.44)	
**Comorbidities** ^**c**^			
Diabetes mellitus	10,110 (12.20)	18,402 (44.43)	< 0.001
Rheumatoid arthritis	764 (0.92)	1,058 (2.55)	< 0.001
Hypertension	14,552 (17.57)	17,644 (42.60)	< 0.001
Hypothyroidism	100 (0.12)	222 (0.54)	< 0.001
Hyperthyroidism	722 (0.87)	1,049 (2.53)	< 0.001
Gout	2,262 (2.73)	8,190 (19.77)	< 0.001
Osteoarthritis	5,193 (6.27)	8,970 (21.66)	< 0.001
Depression	428 (0.52)	358 (0.86)	< 0.001
Carpal tunnel syndrome	1,065 (1.29)	2,149 (5.19)	< 0.001
Distal radial fracture	1,127 (1.36)	518 (1.25)	0.117
Cardiovascular disease	68 (0.08)	116 (0.28)	0.001
Obesity	211 (0.25)	1,290 (3.11)	< 0.001

SD, standard deviation.

Values are expressed as mean ± SD or number (percentage).

^a^Urbanization level was defined at the beginning of the follow-up period.

^b^Insurance amount was measured as the average value during the follow-up period.

^c^Comorbidities were defined before the survival date.

### Association between hyperlipidemia and trigger finger

Table 2 presents the comparison of the trigger finger risks between the hyperlipidemia and control cohorts. The trigger finger incidence was 24.62 per 10,000 person-years in the hyperlipidemia cohort and 6.37 per 10,000 person-years in the control cohort. After adjusting for demographic variables and comorbidities, the hyperlipidemia cohort had a significantly higher adjusted HR for trigger finger (4.03; 95% CI, 3.57–4.55; *P* < 0.001) compared with the control cohort. These findings show that the trigger finger diagnosis risk increased by 303% in people with hyperlipidemia compared with the risk in those without hyperlipidemia. After stratifying the data by sex, trigger finger and hyperlipidemia showed a statistically significant correlation compared with the control cohort. In the hyperlipidemia cohort, the trigger finger adjusted HRs were 4.59 (95% CI, 3.67–5.73; *P* < 0.001) for males and 3.77 (95% CI, 3.26–4.36; *P* < 0.001) for females. The average time from the diagnosis of hyperlipidemia to the occurrence of trigger finger was 5.91 years. Of all patients with trigger finger, 64.26% received surgical intervention. Among patients with trigger finger in hyperlipidemia group, 64.80% underwent surgery. On the other hand, 63.20% of trigger finger patients in the control group received operation.

**Table 2:** Trigger finger incidence and hazard ratios for trigger finger in the hyperlipidemia and control cohorts.

Population	Study cohort	Trigger finger	PY	Rate^a^	Crude HR (95% CI)	Adjusted HR^b^ (95% CI)
**Total**	Control (n = 82,842)	500	784808	6.37	1 (reference)	1 (reference)
	Hyperlipidemia (n = 41,421)	980	398071	24.62	3.86 (3.47–4.30)^‡^	4.03 (3.57–4.55)^‡^
**Female**	Control (n = 38,934)	358	365047	9.81	1 (reference)	1 (reference)
	Hyperlipidemia (n = 19,467)	649	182523	35.56	3.63 (3.19–4.13)^‡^	3.77 (3.26–4.36)^‡^
**Male**	Control (n = 43,908)	142	419761	3.38	1 (reference)	1 (reference)
	Hyperlipidemia (n = 21,954)	331	215548	15.36	4.53 (3.72–5.52)^‡^	4.59 (3.67–5.73)^‡^

CI, confidence interval; HR, hazard ratio; PY, person-years; Rate, incidence rate.

^a^per 10,000 person-years.

^b^Cox regression models were adjusted for age, sex, urbanization level, insurance amount, diabetes mellitus, rheumatoid arthritis, hypertension, hypothyroidism, hyperthyroidism, gout, osteoarthritis, depression, carpal tunnel syndrome, cardiovascular disease and obesity.

^‡^*P* < 0.001.

**Fig 2:**
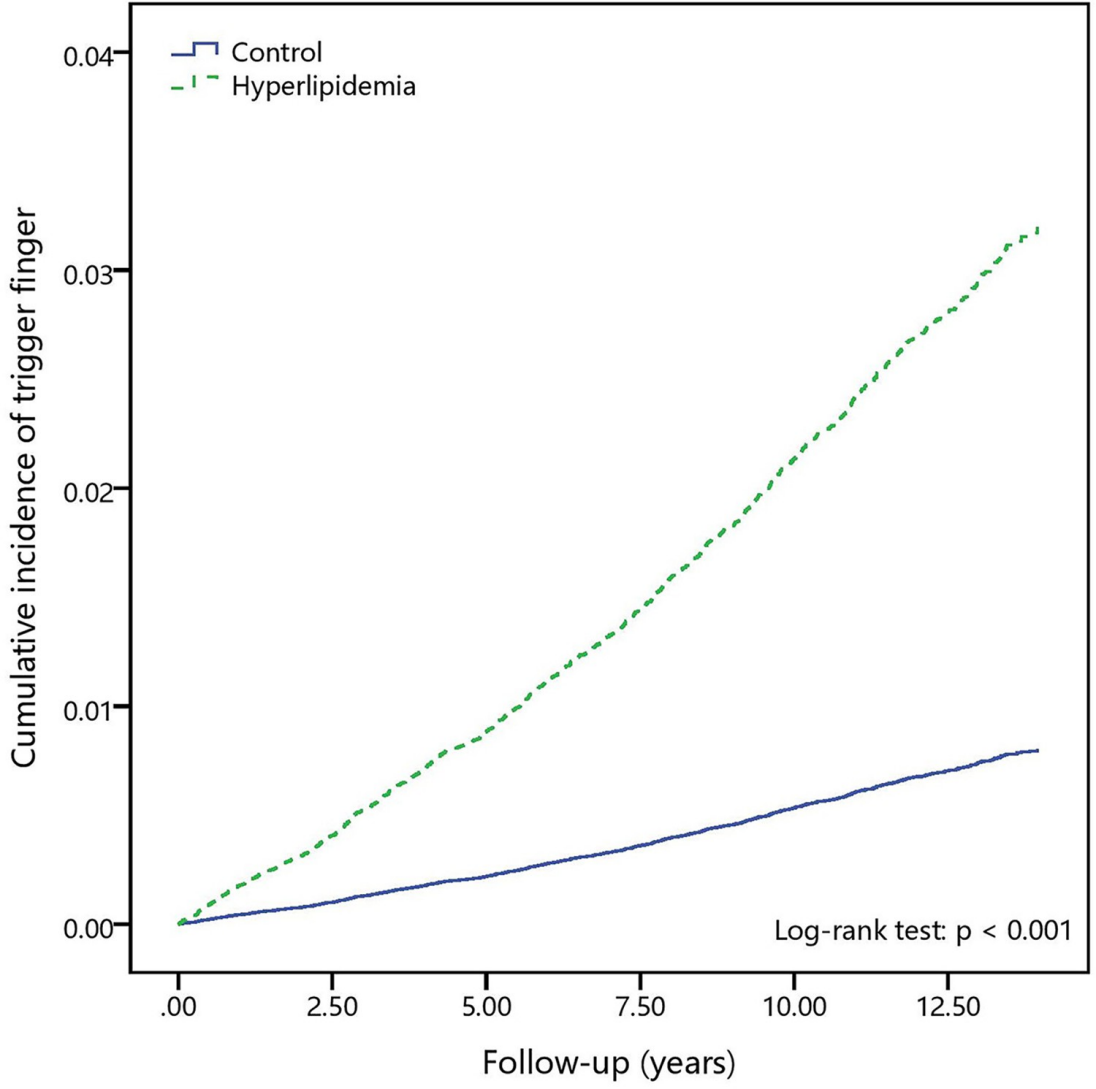
The cumulative incidence curves of trigger finger in individuals with and without hyperlipidemia.

**Fig 3:**
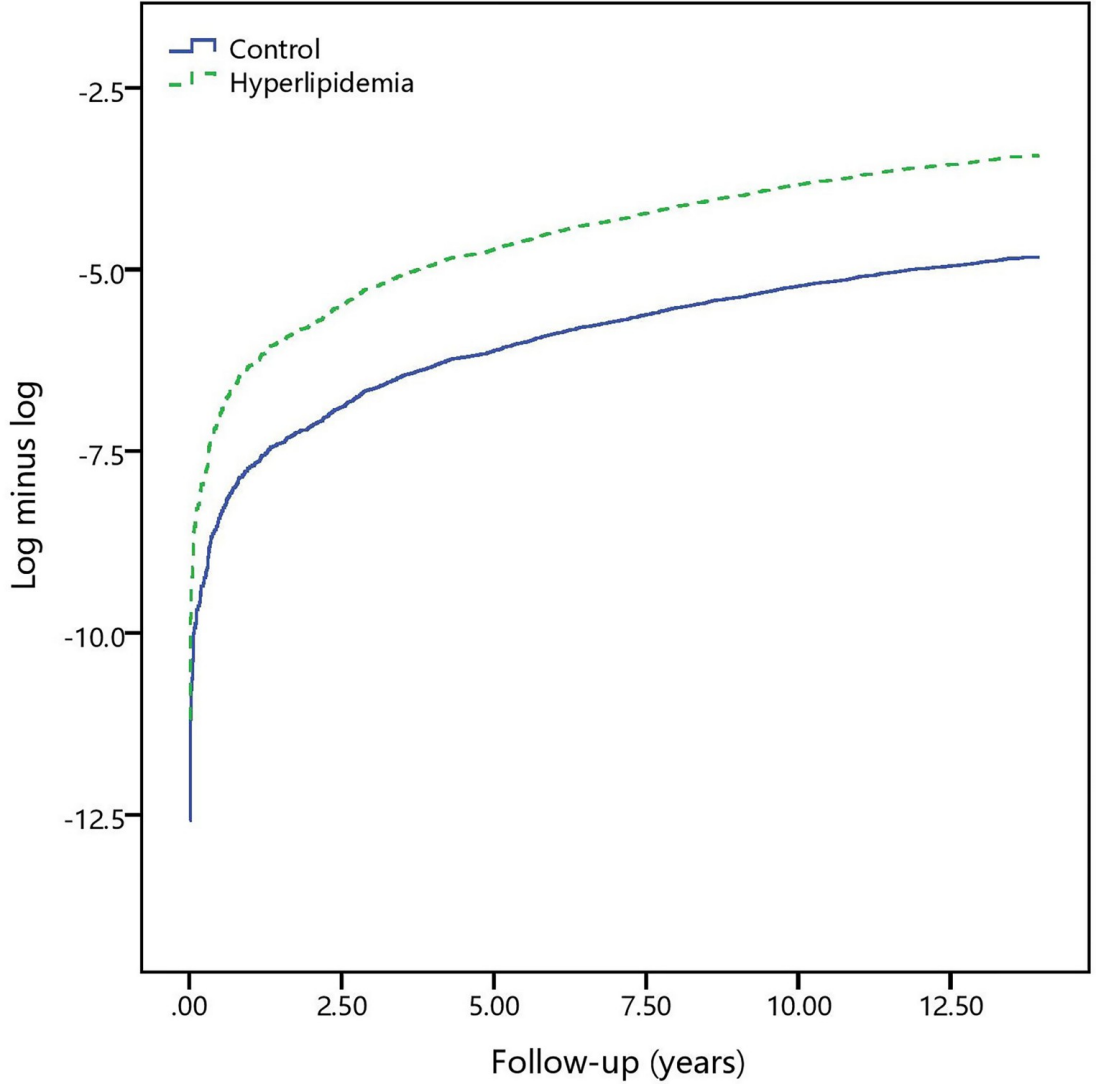
The log minus log survival plot in the control and hyperlipidemia cohorts.

During the follow-up of 13 years, the cumulative trigger finger incidence curves in the hyperlipidemia and control cohorts, evaluated using the Kaplan–Meier method (Fig 2), showed that the incidence was higher in the hyperlipidemia cohort than in the control cohort (log-rank test, *P* < 0.001). Fig 3 shows the log (−log (survival function)) versus survival time for hyperlipidemia, supporting the Cox proportional hazards model.

## Discussion

In this study, we demonstrated that people with hyperlipidemia (both men and women) have a high risk of trigger finger after adjusting for possible comorbidities (diabetes mellitus, rheumatoid arthritis, hypertension, hyperthyroidism, hypothyroidism, gout, osteoarthritis, depression, carpal tunnel syndrome, cardiovascular disease and obesity). People tend to live a sedentary lifestyle in the urban area. Previous study showed that sedentary lifestyle and urbanization are correlated to hyperlipidemia [20, 21]. Insurance amount and financial status would affect the patients’ will to seek medical advice and receive treatment for trigger finger [22, 23]. Therefore, we also adjust urbanization and insurance amount in our models. The adjusted HR was up to 4.03 (95% CI, 3.57–4.55) for people with hyperlipidemia. The findings of our large-scale study suggest that hyperlipidemia is associated with trigger finger.

A retrospective cross-sectional study in Brazil revealed that 80% of patients with trigger finger fulfilled the diagnostic criteria of metabolic syndrome and 60% had dyslipidemia, but the case number was relatively small [24]. This result is consistent with our finding that hyperlipidemia and other metabolic disorders may induce the occurrence of trigger finger. Another retrospective cohort study conducted in Japan revealed that trigger finger is related to incident cardiovascular disease in patients with type 2 diabetes [25].

Hyperlipidemia is a major risk factor for atherosclerosis. Atherosclerosis decreases blood supply and may force tissues to adapt to hypoxic conditions. The histopathological change in trigger finger involves fibrocartilaginous metaplasia between tendon and pulley regions [26]. Degeneration and inflammation in A1 pulleys have also been reported [27]. An increasing number of studies have suggested that fibroblasts undergo fibrocartilage transformation and adaptation under hypoxic conditions [28, 29]. In addition, a high cholesterol level increases the production of reactive oxygen species and induces oxidative stress, which may result in apoptosis and autophagy in tendon-derived stem cells and may cause an inflammatory response [10, 30].

The benefit of this work is that this is the first population-based cohort study to prove the association between hyperlipidemia and trigger finger. The limitation of this study is that the data were extracted from diagnostic codes in a health care insurance system. However, the ICD-9 coding for hyperlipidemia and trigger finger is specific and may decrease the error from data extraction.

Current management of trigger finger includes oral anti-inflammatory agents, splinting, stretching, physical modalities (such as ultrasound, paraffin, etc.), and local steroid injection. Surgical release is needed for patients with refractory disease. The recurrence rate of idiopathic trigger finger after corticosteroid injection has been reported to be 13–56% [31]. High recurrence of trigger finger limits the daily activity of patients and becomes a huge socioeconomic burden. The reduction of the recurrence of trigger finger is important in the fields of physical medicine and rehabilitation.

Hyperlipidemia may be clinically silent for years and is harmful to vascular health gradually. Metabolic screening for patients with trigger finger may help early detection and control of hyperlipidemia. In Taiwan, the surgery costs for trigger finger is about 82 USD per case. Testing for total cholesterol is 2.3 USD and testing for triglyceride is 4 USD. We demonstrated the correlation between hyperlipidemia and trigger finger in this study. In countries without universal healthcare system, screening of lipid profile such as total cholesterol and triglyceride is recommended for people with trigger finger to reduce the burden of cardiovascular diseases. We believe that control of hyperlipidemia by either exercise and diet control or oral lipid-lowering agents may reduce the risk of trigger finger and decrease the use of medical resources.

## Conclusions

This large-scale retrospective population-based cohort study in Taiwan found that hyperlipidemia is correlated to trigger finger. Further observational or experimental studies are needed to validate our hypothesis, and they would be helpful to clarify the pathogenesis of trigger finger.

## List of abbreviations

CI: confidence interval
HR: hazard ratio
ICD-9-CM: International Classification of Disease, 9th Revision, Clinical Modification
LHID: Longitudinal Health Insurance Database
NHIRD: National Health Insurance Research Database
NHI: National Health Institute
